# Association of Nirmatrelvir/Ritonavir Treatment and COVID-19-Neutralizing Antibody Titers in a Longitudinal Health Care Worker Cohort

**DOI:** 10.1093/ofid/ofad625

**Published:** 2024-02-13

**Authors:** Slade Decker, Shaoming Xiao, Carly Dillen, Christina M Schumacher, Aaron M Milstone, Matthew Frieman, Amanda K Debes

**Affiliations:** Johns Hopkins University School of Medicine, Baltimore, Maryland, USA; Johns Hopkins University School of Medicine, Baltimore, Maryland, USA; Center for Pathogen Research, Department of Microbiology and Immunology, University of Maryland School of Medicine, Baltimore, Maryland, USA; Johns Hopkins University School of Medicine, Baltimore, Maryland, USA; Johns Hopkins University School of Medicine, Baltimore, Maryland, USA; Johns Hopkins University Bloomberg School of Public Health, Baltimore, Maryland, USA; Center for Pathogen Research, Department of Microbiology and Immunology, University of Maryland School of Medicine, Baltimore, Maryland, USA; Johns Hopkins University Bloomberg School of Public Health, Baltimore, Maryland, USA

**Keywords:** antibodies, antivirals, COVID-19, humoral immune response, immunology, longitudinal cohort, NMV/r

## Abstract

Nirmatrelvir/ritonavir (NMV/r) is used for the treatment of coronavirus disease 2019 (COVID-19) infection. However, rebound COVID-19 infections can occur after taking NMV/r. We examined neutralizing antibodies to the severe acute respiratory syndrome coronavirus 2 spike protein before and after infection in people who did and did not take NMV/r to determine if NMV/r impedes the humoral immune response.

In 2021, the US Food and Drug Administration granted Emergency Use Authorization to nirmatrelvir/ritonavir (NMV/r) for the treatment of mild to moderate coronavirus disease 2019 (COVID-19) infection. Soon after, the United States began providing antiviral medication at no cost to patients to reduce COVID-19-related hospitalizations and mortality. Reports then emerged of rebound COVID-19 infections, defined as detectable viremia and/or symptoms, 2–8 days after completing NMV/r treatment [1–3]. One hypothesis to explain post-treatment rebound infection is that early viral suppression reduces the immune response, including antibody production, thereby predisposing to recurrent viremia and symptoms [4–6]. Prior studies have found that for severe acute respiratory syndrome coronavirus 2 (SARS-CoV-2) and other viruses, early viral suppression (ie, within 2–5 days of infection) can weaken immune response to infection [4, 5]. Our objective was to examine neutralizing antibodies to the SARS-CoV-2 spike protein before and after infection in people who did and did not complete an NMV/r treatment course to elucidate whether NMV/r is associated with a decreased humoral immune response to SARS-CoV-2.

## METHODS

### Study Population

Health care workers (HWs) were consented into an ongoing seroprevalence cohort beginning in June 2020. Approximately every 2–6 months, HW provided serum samples and completed surveys that captured data including COVID-19 infection dates and NMV/r use. We measured changes in neutralization antibody levels using pre-/postinfection serum samples from each individual meeting inclusion criteria and compared changes in neutralization antibody levels by NMV/r use. HWs who received NMV/r (treated) were matched to an HW who did not (untreated). Inclusion criteria included (1) COVID-19 infection (positive polymerase chain reaction or antigen test), (2) no prior infection or receipt of vaccine dose within 90 days of pre-infection serum collection, (3) had serum collected and stored within 90 days before infection, and (4) had serum collected and stored at least 10 days postinfection. Treated HWs were matched to untreated HWs who had (1) an infection date +/−14 days of a treated HW and (2) dates of both the pre- and postinfection serum collection that were within +/−7 days of the treated HW serum collection. This study was approved by the Johns Hopkins University institutional review board.

### Neutralization Assay

Neutralizing antibody titers (NTs) to the spike protein of SARS-CoV-2 (WA-1) for pre-NMV/r and post-NMV/r (treated) samples were compared with untreated controls to determine if NMV/r had a measurable response on neutralizing antibody response to infection. For the neutralization titer assays, serum samples were heat-inactivated at 56°C for 30 minutes to remove complement and allowed to equilibrate to room temperature before processing for neutralization titer. Samples were diluted in duplicate to an initial dilution of 1:10 followed by 1:2 serial dilutions, resulting in a 12-dilution series, with each well containing 100 mL. All dilutions were performed in DMEM (Quality Biological) and supplemented with 10% (v/v) fetal bovine serum (heat inactivated, Sigma), 1% (v/v) penicillin/streptomycin (Gemini Bio-products), and 1% (v/v) L-glutamine (2-mM final concentration, Gibco). Dilution plates were then transported into the BSL-3 laboratory, and SARS-CoV-2-GFP inoculum was added to each well, to result in a multiplicity of infection (MOI) of 0.01 upon transfer to titration plates. A nontreated, virus-only control and a mock infection control were included on every plate. The sample/virus mixture was then incubated at 37°C (5.0% CO2) for 1 hour before transferring to 96-well flat-bottom plates with confluent Vero-TMPRSS2 cells. Titer plates were incubated at 37°C (5.0% CO2) for 24 hours after cells were fixed with 10% neutral buffered formalin (Sigma) for at least 1 hour at 4°C as per BSL3 standard operating procedure. Plates were removed from the BSL3 and stained with Hoechest 33342 (Thermo Scientific) diluted 1:2000 in phosphate-buffered saline (PBS) for 10 minutes. Cells were washed with PBS and imaged with a Celigo high-content imager (Nexcelom).

### Statistical Analyses

To measure the differences in the relative change in antibody response pre- and postinfection among matched pairs, we calculated the ratio of the half maximal inhibitory concentration (IC50) between matched participants pre- and, separately, postinfection. The IC50 ratios were then compared using the Wilcoxon signed rank test. Statistical significance was defined as *P* < .05. Analyses were performed in R, version 4.2.2 (R Foundation for Statistical Computing, Vienna, Austria).

## RESULTS

Of 1353 HWs who reported COVID-19 infection, 65 reported NMV/r use and 32 met inclusion criteria, of whom 21 could be matched to a control. We performed neutralization assays on serum from 21 HWs treated and 21 untreated with NMV/r. Of the 42 participants, the majority were female (86%) and White (90%). Treated participants were older (median [interquartile range {IQR}], 50.9 [44.6–62.0] years) and less frequently female (78% vs 94%) than untreated participants (median [IQR], 35.5 [32.6–40.9] years). Treated and untreated participants did not substantially differ by preexisting conditions or prior exposure to SARS-CoV-2 spike protein (due to infection and/or vaccine) (Supplementary 1).

**Figure 1.**
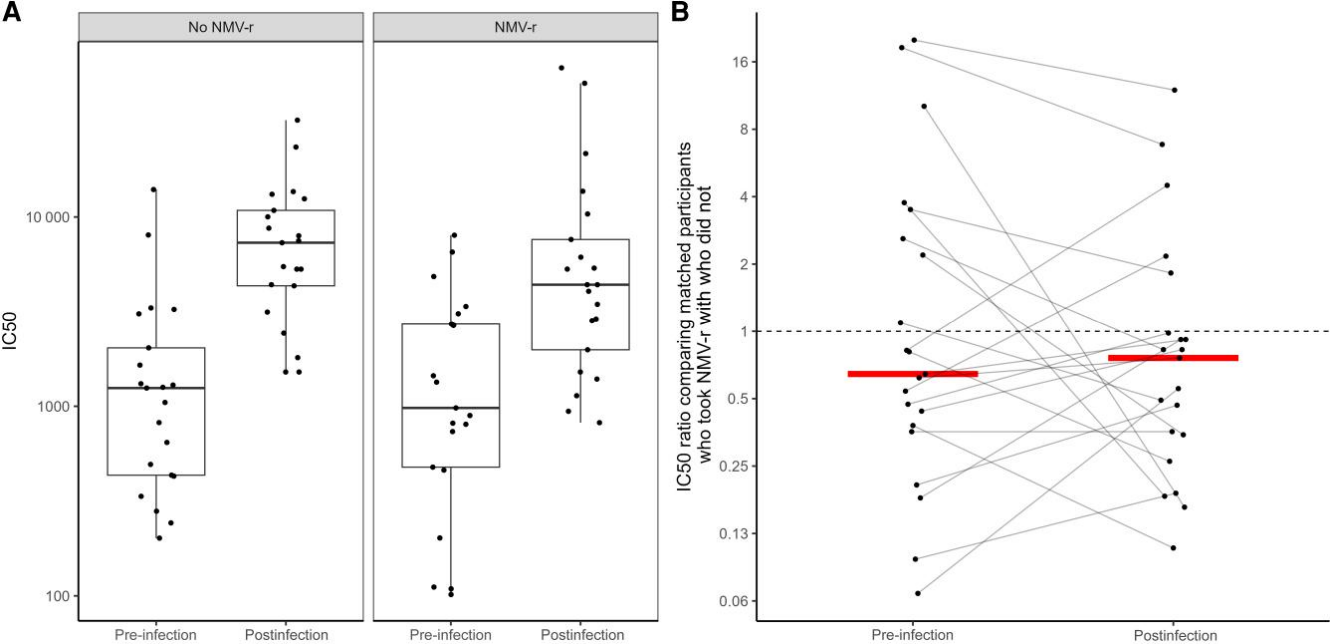
Antibody response to SARS-CoV-2 by NMV/r use among unmatched and matched pairs. A, IC50 pre-infection and postinfection among unmatched participants who took NMV/r (n = 21) and those who did not (n = 21). Treated participants were not matched to their paired untreated controls in this panel. IC50 values are displayed using a log scale of 10. B, IC50 ratio of matched paired participants (linked by gray lines) who took NMV/r vs those who did not, measured between pre-infection and postinfection serum samples (n = 21). The dashed line represents an equivalent change in IC50 value (IC50 ratio = 1) among matched pairs pre-compared with postinfection. The red lines represent the median for each group. IC50 ratio values are displayed using a log scale of 2. Abbreviations: IC50, half maximal inhibitory concentration; NMV/r, nirmatrelvir/ritonavir; SARS-CoV-2, severe acute respiratory syndrome coronavirus 2.

The pre-infection and postinfection IC50 in the treated group were lower than in the untreated group (Figure 1A). The median (IQR) fold change in NTs in the treated group pre- and postinfection was 3.6 (1.3–11.2) (Table 1). The median (IQR) fold change of the NTs in the untreated group pre- and postinfection was 5.1 (4.0–12.8) (Table 1). Of the treated and untreated groups, 67% and 61%, respectively, had an NT ≥800 pre-infection, and 100% of both groups had an NT ≥800 postinfection. The ratios of IC50 between the treated and untreated groups pre- and postinfection were similar (median, 0.64 and 0.76, respectively; *P* = .7) (Figure 1B).

**Table 1.** Live Virus Neutralizing Antibody Titers Against the Vaccine Strain (WA-1)

Study Group	Pre, Median (IQR)	Post, Median (IQR)	Fold Change, Median (IQR)
NMV/r	980.0 (477.3–2720.3)	4394.0 (1986.4, 7608.4)	3.6 (1.3–11.2)
No NMV/r	1247.5 (433.0–2032.7)	7316.0 (4335.0–10 834.0)	5.1 (4.0–12.8)

Abbreviations: IQR, interquartile range; NMV/r, nirmatrelvir/ritonavir.

## DISCUSSION

This study demonstrates that taking NMV/r after COVID-19 infection is not associated with a diminished NT against WA-1. Our results show that the ratios between pre-infection and postinfection NTs for the treated and untreated groups were nearly identical, with median ratios in IC50 values of 0.64 and 0.76, respectively. Additionally, the fold change in NTs between the treated and untreated groups had overlapping IQRs, with fold-change IQRs of 1.3–11.2 and 4.0–12.8, respectively. While this study demonstrates that there is no significant difference in postinfection NTs between treated and untreated groups, it highlights that despite high pre-infection NTs, both groups still got infected with SARS-CoV-2. Other researchers have attempted to create clarity on the NT threshold of protection, but it remains poorly defined [7].

Our goal was to see if NMV/r has an impact on host humoral immune response during COVID-19 infection, and we assessed this by comparing the increase of neutralizing antibodies pre- with postinfection. A recent publication accessing immunoglobulin G (IgG) and Omicron-specific neutralizing antibodies in individuals taking NMV/r using only postinfection data reported similar findings, including similar neutralizing antibody levels when taking the medication and when not, with an overall conclusion that NMV/r treatment does not impact adaptive immune response [8]. Our results suggest that concern about early viral suppression reducing immune response to infection should not be a barrier or deterrent to taking NMV/r.

We believe the observed difference in age may be due to indication bias for treatment based on age; older age has been associated with higher risk for severe COVID-19 infection, leading to an increase in older individuals taking NMV/r. The difference in pre-infection and postinfection NTs between the treated and untreated groups may be attributed to the age differences across study groups. Prior work has found that compared with older people, younger people produce a higher antibody response to COVID-19 [9, 10].

The limitations of this study include a small sample size (n = 42) and a demographically homogenous cohort of HWs, which limits generalizability, although our sample size is significantly larger than those used in a similar prior study [8]. Second, we only performed NTs on WA-1 SARS-CoV-2. Despite the antigenic differences between original SARS-CoV-2 and its numerous variants, including Omicron, we tested only the wild-type WA-1 strain as that is an identical match to the vaccine strain. Our study population consisted entirely of vaccinated individuals, so using the same strain as the vaccine would result in the most robust responses. However, we cannot exclude the possibility that variant-specific NTs may vary between untreated and treated groups.

The results of this study show that NMV/r medication does not have a significant impact on humoral immune response against WA-1 postinfection. This finding should reassure those infected with COVID-19 that they can take NMV/r without negatively impacting their humoral immune response. More work is needed to break down barriers that have prevented the acceptance of this drug as an important treatment option for COVID-19. This study examined immune response relatively soon after infection; future research is needed to examine the impact of rebound COVID-19 infections on neutralizing antibody titers and the rate of anti-SARS-CoV-2 antibody degradation in individuals who take NMV/r compared with those who do not over longer time periods postinfection. Research is also needed to assess variant-specific NTs. Our findings suggest that those at high risk can take NMV/r to reduce the severity and length of mild to moderate COVID-19 infection without detriment to their postinfection humoral immune response.

## Supplementary Material

ofad625_Supplementary_Data

## Supplementary Data

Supplementary materials are available at *Open Forum Infectious Diseases* online. Consisting of data provided by the authors to benefit the reader, the posted materials are not copyedited and are the sole responsibility of the authors, so questions or comments should be addressed to the corresponding author.
