# Supplementary material for: Association of Nirmatrelvir/Ritonavir Treatment and COVID-19-Neutralizing Antibody Titers in a Longitudinal Health Care Worker Cohort
Source: Open Forum Infect Dis. 2024 Feb 13;11(2):ofad625. doi: 10.1093/ofid/ofad625 (PMC10863641; doi:10.1093/ofid/ofad625)
Supplement: ofad625_Supplementary_Data [file ofad625_supplementary_data.docx]

**Supplemental 1.** Demographics and clinical factors by Nirmatrelvir/Ritonavir (NMV/r) treatment status among healthcare workers infected with SARS-CoV-2. Spike exposures include those due to prior infections or vaccine

|  | NMV/r | No NMV/r |
| --- | --- | --- |
| N | 21 | 21 |
| Age: median (IQR) | 50.9 (44.6 – 62.0) | 35.5 (32.6 – 40.9) |
| Sex: Female (%) | 16 (76) | 20 (94) |
| Chronic lung disease (%) | 1 (5) | 0 |
| Diabetes (%) | 2 (10) | 0 |
| Hypertension (%) | 4 (20) | 2 (10) |
| Obesity (%) | 5 (24) | 3 (14) |
| Any symptoms at diagnosis (%) | 16 (76) | 20 (94) |
| Vaccine type (%)  Moderna  Pfizer  Unspecified | 13 (20)  51 (80)  0 | 16 (25)  46 (72)  2 (3) |
| Days from last spike exposure to date of pre-infection sample: median (IQR) | 168 (158 - 215) | 192 (153 - 248) |
| 2 prior spike exposures (%) | 0 | 1 (5) |
| 3 prior spike exposures (%) | 19 (90) | 18 (85) |
| 4 prior spike exposures (%) | 2 (10) | 2 (10) |
